# Translating evidence-based guidelines into practice: a survey of practices of commissioners and managers of the English stop smoking services

**DOI:** 10.1186/1472-6963-12-121

**Published:** 2012-05-23

**Authors:** Máirtín S McDermott, Heather Thomson, Robert West, Jennifer AM Kenyon, Andy McEwen

**Affiliations:** 1 NHS Centre for Smoking Cessation and Training (NCSCT), Department of Educational, Clinical and Health Psychology, University College London, London, WC1E 6BT, UK; 2 NHS Leeds, Strategy and Commissioning Directorate, Suite C, Ground Floor, North West House, West Park Ring Road, Leeds, LS16 6QG, UK; 3 Cancer Research UK Health Behaviour Research Centre, Department of Epidemiology and Public Health, University College London, London, WC1E 6BT, UK

## Abstract

**Background:**

The English National Health Service’s (NHS) Stop Smoking Services (SSSs) constitute one of the most highly developed behavioural support programmes in the world. However, there is significant variation in success rates across the approximately 150 services, some of which may be due to variation in practice. This study aimed to assess these differences in practice.

**Methods:**

Two online surveys were administered. All commissioners (people who purchase services for the NHS) and managers (those who run the services) of NHS SSSs in England were invited to participate. Items included details of current practices and services provided, what informed the commissioning of SSSs, what targets were included within service specifications and whether the types of treatment model to be delivered were specified.

**Results:**

Both surveys had a response rate of 35%, with 50 commissioners and 58 managers participating. There were no significant differences between the characteristics of the Primary Care Trusts (PCTs) from which commissioners and managers responded to this survey and those PCTs from which there was no response. Managers reported that the treatment model most frequently offered by SSSs was one-to-one (98%). A total of 16% of managers reported that some approved medications were not available as first-line treatments. Just over one third (38%) of commissioners reported consulting national guidelines or best evidence to inform local commissioning. Almost one third (30%) of commissioners reported that they specified the types of stop smoking interventions to be delivered by the providers.

**Conclusions:**

A substantial part of commissioning of Stop Smoking Services in England appears to take place without adequate consultation of evidence-based guidelines or specification of the service to be provided. This may account for at least some of the variation in success rates.

## Background

In 1999, the UK government’s tobacco control strategy, published in the White Paper ‘Smoking Kills’[1], established the treatment of smokers as an integral part of the effort to reduce smoking prevalence [2]. This led to the formation of the English National Health Service’s (NHS) Stop Smoking Services (SSSs). The SSSs were a world first and have come to be considered the ‘blue-print’ for smoking cessation programmes throughout the world. Each offers smokers a combination of evidence-based behavioural and pharmacological treatments paid for or subsidised through taxation. The SSSs have been shown to be both effective [3,4] and cost-effective [5] and have treated over three million smokers thus far [6,7].

Each SSS is organised independently within its local Primary Care Trust (PCT), of which there were 152 at the time the current study was conducted. PCTs are organisations currently responsible for identifying local priorities and commissioning healthcare, including smoking cessation, to serve the needs of their local population within specific budgets. Services are then free to configure their practices as appropriate to meet local priorities and targets, with the expectation they will reflect evidence-based guidelines from national bodies such as the National Institute for Health and Clinical Excellence (NICE) [5] and the Department of Health (DoH) [6].

A process is normally followed when commissioning SSSs that includes the identification of local need using a range of qualitative and quantitative data, followed by an analysis of how current service provision meets that need. Priorities are then determined which would inform service specifications and performance indicators, against which providers would be performance managed. Whilst such processes may allow services to be responsive to local needs, they may also have contributed to a drift away from the evidence-base. Initially, it was recommended by DoH that the primary stop smoking intervention offered to smokers should comprise group support plus nicotine replacement therapy (NRT), with weekly meetings covering six weeks. The provision of one-to-one support was also specified where appropriate such as in cases where smokers could or would not attend groups [8]. However, it was found that within a few years of their establishment SSSs were increasingly providing one-to-one sessions [8]. By 2009/2010, only 6% of smokers setting a quit date with the services did so in a group [9]. Variability in practice may also have contributed to the wide variation in success rates across SSSs. In 2009/2010 four-week biochemically validated quit rates ranged from 3–58%, with an average of 34% [9]. Rates of biochemical validation (by expired air carbon monoxide) provide explanation for some of this variation, yet even when self-reported (non-verified) quits are considered, the variation remains wide, from 31–70%, with an average of 49% [9].

Current guidelines recommend that smokers should have a choice between a number of different pharmacological and behavioural interventions, whilst acknowledging that there is evidence of superior efficacy for some. Three medications with evidence of effectiveness are recommended: Nicotine Replacement Therapy (NRT), bupropion (Zyban®) and varenicline (Champix® in the UK and Chantix® in the USA). There is evidence of superiority of varenicline or combination NRT (i.e. simultaneously using two different types of NRT) over other forms of pharmacology or no medication in both clinical trials [10-12] and routine clinical practice [13]. Guidelines also recommend that medication is used in combination with behavioural support, usually in the form of individual counselling or in groups, both of which are also of proven efficacy in clinical trials [14,15], with evidence from routine clinical practice indicating that group treatment is more effective than one-to-one support [13,16,17]. The ability of SSSs to deliver high quality evidence-based interventions also depends on their stop smoking practitioners (SSPs) being trained to a minimum standard and being able to demonstrate and maintain the knowledge and skills for effective job performance. Current guidelines also recommend that interventions should be delivered by practitioners who have had training and supervision that complies with the NHS Centre for Smoking Cessation and Training (NCSCT) Training Standard [18].

However little is known, either about the extent to which these guidelines are adhered to in practice, or about the processes determining how they are applied. The surveys reported in this paper were carried out as part of a programme of research conducted at the NHS Centre for Smoking Cessation and Training (NCSCT). One aim of the NCSCT is to establish what constitutes best practice in treatment to aid smoking cessation, and to develop and implement assessment and training to ensure that all practitioners possess the competences necessary to deliver effective interventions (see http://www.ncsct.co.uk). The aim of the current study, therefore, was to describe the current practices and experiences of commissioners (i.e. those individuals who are responsible for specifying and commissioning services to meet local need and monitoring performance of providers) and managers (i.e. those responsible for the day to day planning and delivery of SSSs) to identify possible factors within the contracting of services that could account for the variation in clinical outcome across England and to investigate any association between these factors and quit rates at commissioned SSSs.

## Methods

### Study design

Two online surveys were administered.

### Participants and survey administration

Both surveys were available via a hyperlink sent out in an electronic flier to all commissioners and managers of SSSs in England. The NCSCT maintains databases of commissioners and managers of SSSs and this information was verified prior to launching the 2010 survey. Reminders were sent at 10 and 20 days after the initial contact, with a final reminder sent three days preceding the survey’s close. The commissioners’ survey was open between 22nd November and December 24th 2010. The managers’ survey was open between 26th November and December 24th 2010. The current study was classed as a service evaluation of the current SSSs by the University College London Research Ethics Committee and so was exempt from ethical review.

### Measures

The commissioners’ survey contained 28 items covering a range of topics (see Additional file 1: Appendix A). The survey first asked for contact, basic demographic and employment details. Following this, the survey asked about the commissioning of stop smoking services, specifically whether commissioning was informed by their PCT’s priorities, what targets they included within their service specifications, whether they took various elements of support into account in their commissioning framework and whether they specified the types of treatment models to be delivered by providers. We also asked whether service specifications included the availability of all NICE-recommended medications as first-line treatments, and the level of training that practitioners were expected to undertake.

Managers completed a 29-item questionnaire survey (see Additional file 1: Appendix B) which also first asked for contact, basic demographic and employment details. Items then covered commissioning, specifically the targets commissioners include within their service specifications, whether commissioners specify the settings or treatment models they should run and managers’ relationship with their commissioner. Items were also asked regarding the services provided by managers and the settings in which their services were provided.

A combination of closed and open questions was used. Where respondents were presented with a range of categories to choose from, an ‘other’ option was also included in order to invite the widest range of responses. Drafts of the questionnaire were completed by NCSCT personnel (including a current commissioner, ex-SSS manager and two practitioners) for the purposes of refinement prior to launching online.

Both commissioners and managers were asked to report their employing PCT. This was then used to link survey responses to data characterising the area in which their PCT is located (Index of Multiple Deprivation (IMD) [19]) and outcome and throughput data for the local SSS (self-reported and CO-validated quit rates, and number of smokers setting a quit date). The IMD is an overall, continuous measure of multiple deprivation experienced by people living in an area and is calculated by combining scores from seven distinct domains: Income Deprivation, Employment Deprivation, Health Deprivation and Disability, Education Skills and Training Deprivation, Barriers to Housing and Services, Living Environment Deprivation, and Crime [19]. SSS data was taken from official NHS Stop Smoking Service statistics collected between April 2010 and March 2011 [7].

### Procedure

In total, 68 responses were recorded to the commissioners’ survey. Of these, nine contained no data and four supplied contact details only, these entries were all excluded. Three commissioners had one duplicate entry and one commissioner had two duplicate entries; in these cases only the most complete set of answers was retained. For the managers’ survey, a total of 85 responses were recorded. Of these nine did not contain any data and 11 supplied contact details only, these entries were excluded. Five managers had one duplicate entry and one manager had two duplicate entries, only the most complete set of answers was retained. Therefore 50 commissioners (effective response rate = 35.2%, 50/142) and 58 managers (effective response rate = 35.4%, 58/164) completed the survey and it is on this data that the findings are reported.

### Data analysis

Data were transferred to SPSS (Version 14) where they were anonymised, coded and analysed. Basic descriptive statistics were used to analyse participants’ responses. Rates of missing data varied between 0% and 24% for commissioners’ responses and 0% and 28% for managers. No attempt was made to estimate missing values. Respondents’ free-text responses were analysed using a content analysis approach whereby text was analysed by looking at the frequency of matching responses and converted into categorical variables for analysis. Categorical variables were analysed using basic descriptive statistics. Differences between responding and non-responding PCTs in abstinence rates, service throughput and IMD score were investigated using independent samples t-tests, as were differences in SSS abstinence rates.

## Results

### Respondent characteristics

Commissioner and manager demographic and employment details and details of their PCTs can be seen in Table 1. A total of 23 PCTs provided data from both commissioner and manager.

**Table 1:** Commissioner and manager demographic, employment and PCT/SSS-level details

		**Commissioners (n = 50)**^**a**^	**Managers (n = 58)**^**a**^
**Individual-level characteristics**			
Gender	Female: %(n)	74 (37)	78 (35)
Mean length of time commissioning health services (months)	Mean (SD, Range)	44.1 36.2, 6–180	-
Mean length of time commissioning/managing stop smoking services (months)	Mean (SD, Range)	39.6 (33.4, 3–132)	44.8 (37.15, 6–144)
Is commissioning/managing stop smoking services your sole responsibility?	Yes: % (n)	14 (5)	24 (14)
% of current role spent commissioning/managing stop smoking services	Mean (SD, Range)	41.9 (27.1, 5–100)	83.4 (22.7, 20–100)
N other services commissioned/managed	Mean (SD, Range)	2.9 (1.4, 1–6)	1.6 (0.7, 1–3)
N WTE ‘Specialist’ SSPs	Mean (SD, Range)	-	6.9 (5.8, 0–30)
N active community SSPs	Mean (SD, Range)	-	118.3 (95.3, 0–320)
**PCT-level characteristics**		**(n = 48)**^**b**^	**(n = 55)**^**b**^
Self-reported quit rate	Mean % (SD, Range)	50.1 (8.6, 32.8–68.9)	48.0 (8.7, 29.5–68.9)
CO-validated quit rate	Mean % (SD, Range)	35.9 (8.8, 14.8–56.6)	33.7 (10.6, 4.8–56.6)
Number of smokers setting a quit date	Mean (SD, Range)	5764.5 (3212.4, 1619–16858)	5527.9 (2590.7, 1619–14515)
IMD Score ^**c**^	Mean (SD, Range)	24.1 (8.8, 11.3–43.5)	25.2 (9.5, 10.0–43.5)
N setting a quit date per WTE specialist SSP	Mean (SD, Range)		1172.3 (941.1, 275.2–4568.0)
N setting a quit date per ‘active’ community SSP	Mean (SD, Range)		232.9 (763.0, 16.4–4568.0)

^a^ % may not add up to 100% due to rounding and missing data.

^b^ Some PCTs had multiple responding commissioners/managers.

^c^ Higher scores indicate greater deprivation.

The PCTs from which the samples of commissioners and managers participating in the current study were drawn did not differ from those who did not participate in a number of important variables. There were no differences between PCTs with participating commissioners and those where commissioners did not participate, in self-reported (responder mean = 50.1%, vs. non-responder mean = 49.2%, t(150) = −0.63, p = .53) or CO-validated quit rates (35.9% vs. 34.5%, t(150) = −0.82, p = .41), number setting a quit date at the SSS (5764.5 vs. 5073.95, t(150) = −1.38, p = .17) and PCT IMD score (24.1 vs. 23.4, t(150) = −0.48, p = .63). Similarly, there were no differences between PCTs with responding managers and those where managers did not participate in self-reported (responder mean = 48.0%, vs. non-responder mean = 50.3%, t(150) = 1.78, p = .08) or CO-validated quit rates (33.7%, vs. 35.7, t(150) = 1.19, p = .24), number setting a quit date at the SSS (5527.9 vs. 5158.3, t(150) = −0.76, p = .45) and PCT IMD score (25.2 vs 22.8, t(150) = −1.71, p = .09).

Managers reported employing an average of 6.9 (SD = 5.8, range = 0–30) Whole Time Equivalent (WTE) *‘Specialist’* SSPs (i.e. SSPs employed directly by the SSS specifically to deliver stop smoking interventions). In addition, 84% of managers (n = 48) stated that their SSS was responsible for the support or performance management of *‘Community’* SSPs (i.e. SSPs, who deliver support for the SSS as part of or in addition to their main role, typically as practice nurses or community pharmacists), with 245 (SD = 335.0, range = 1–2000, median = 140) community SSPs being performance managed on average. Managers estimated that 68% (SD = 23.5, Range 0–100) of these other practitioners were ‘active’, which was defined as having supported over five clients in the last six months and returned monitoring forms (i.e. data on the numbers of smokers entering treatment, setting a quit date and their outcome (e.g. quit, relapsed, lost to follow-up). Based on this estimate, the mean number of ‘active’ community SSPs can be seen in Table 1.

### Commissioning of stop smoking services

**Figure 1:**
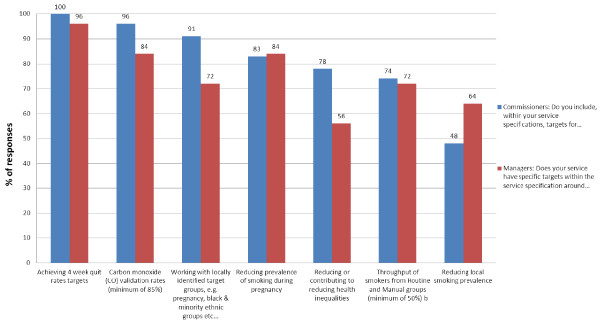
**Targets included by commissioners within their service specifications and within managers’ service specifications for their service**^**a**^**(Figure **1**(Revised).doc).**

Figure 1 gives details for targets commissioners include within their service specifications and managers had within the service specifications for their service, restricted to those commissioners and managers who responded from the same PCT. The majority (88%, n = 44) of managers said that they had regular arranged meetings with their commissioner and 94% (n = 44) said that they had a good relationship with their commissioner.

The vast majority, 92% (n = 44) of commissioners said that SSSs were commissioned from an NHS provider organisation and 49% (n = 23) that SSSs were commissioned from a single provider organisation. A total of 78% (n = 35) of commissioners stated that the commissioning of SSSs was based on a local needs assessment. In free-text responses, the most frequently cited source of information contained in the assessment was smoking prevalence (67%, n = 22), followed by information on demographics (36%, n = 12), mortality and morbidity (30%, n = 10) and economic deprivation (30%, n = 10). When asked what informed local commissioning beyond the local needs assessment, again using free-text responses, the most frequently cited source of information was best evidence or national guidance (e.g. from DoH, NICE, 38%, n = 15), user experience (28%, n = 11) followed by the annual SSS report or other survey data (15%, n = 6) and local knowledge (13%, n = 5).

### Services provided

A total of 70% (n = 33) of commissioners said that they had developed a commissioning framework that takes various types of stop smoking interventions (e.g. very brief advice through to intensive support) and different settings for provision into account. The same proportion of commissioners (70%, n = 35) reported that they specified the types of stop smoking interventions to be delivered by the providers.

In contrast, only 24% (n = 13) of managers stated that the commissioner of the SSS specified the settings in which the service should run and 36% (n = 19) stated that the commissioner of their SSS specified the types of stop smoking interventions to be offered. A total of 17% (n = 8) of commissioners and 15% (n = 7) of managers said that not all recommended medications (i.e. NRT, bupropion & varenicline) were available as first-line treatments. Table 2 shows the treatment models managers’ report their services offer, the settings in which their treatments run, and the availability of medications at their SSS.

**Table 2:** Treatments models offered and settings in which services run according to SSS managers (n = 58)

**Treatment models offered**^**a**^	**% (n)**
One-to-one appointments	98 (57)
Telephone advice/counselling	90 (52)
Home visits	83 (48)
Self-help materials	79 (46)
One-to-one drop-in sessions	78 (45)
Closed group programmes	69 (40)
Rolling group programmes	69 (40)
Peer led sessions	14 (8)
**Settings in which services run**^**a b**^	
Primary care settings	93 (54)
Workplaces	90 (52)
Secondary health care settings (e.g. hospitals)	88 (51)
Voluntary sector/Local Authority premises	86 (50)
Pharmacies	86 (50)
Commercially rented venues	60 (35)
Central base exclusive to SSS	43 (25)
**Availability of medications (first line)**	
Nicotine Replacement Therapy	100 (49)
Varenicline (Champix)	86 (42)
Bupropion (Zyban)	84 (41)
All medications	84 (41)

^**a**^ Participants could choose more than one category.

^**b**^ Groups can be ‘open (rolling)’ or ‘closed’; open groups are open to new members at each session, i.e. individuals within the same group will be at different points in their quit attempt and have different quit dates. A closed group in contrast is a group in which all members start their quit attempt together and new members cannot join after the first meeting. Drop-ins differ from one-to-one support in that they operate without fixed appointments and number and timings of sessions are less fixed (3).

The majority (80%, n = 36) of commissioners reported that service specifications for smoking stated the level of training that practitioners are expected to achieve. The most frequently cited level to be achieved was in line with NCSCT guidelines [18] (or HDA standard as was required formerly [20]) (53%, n = 17).

## Discussion

There was little evidence of commissioners routinely referring to evidence-based guidelines when commissioning services. Whilst an explicit question was not asked regarding references to guidelines in service specifications, based on free-text responses just over one third of commissioners mentioned that best evidence or guidelines from national bodies such as NICE or the Department of Health were used to inform the commissioning of local SSSs. This is in spite of commissioners being prompted to refer to the evidence base within the NHS Standard Community Services Contract [21].

It is possible, in recognition of the knowledge and experience of SSS managers that many commissioners would not explicitly specify that stop smoking interventions should be based on current evidence, focussing instead on the targets to be met, with the expectation that guidelines would be adhered to. There may also be a failure of communication between commissioners and managers as to the content of service specifications. Commissioners and managers from the same PCTs did not appear to be in agreement over the targets reported in service specifications. Although this may be also be due to only managers from ‘core’ Stop Smoking Services having been invited to participate, whereas commissioners may have responded taking into account multiple service providers, such as those deliver smoking cessation locally, e.g. services commissioned from Primary Care providers such as GP practices or pharmacists. In addition over two thirds of commissioners reported that they specified the types of stop smoking interventions to be delivered by the providers, whilst only one third of managers indicated that this was the case. It is possible that managers might have interpreted the word ‘specify’ as implying that the requirements of commissioners are stated explicitly without consultation. Given the vast majority of managers reported having a good relationship with their commissioner, this might explain why so few endorsed this item. However, the NHS Standard Community Services Contract [21] does prompt specification of service delivery, and so whatever the decision making process, commissioners will either specify the types of intervention to be included in the service provided or not, and so this discrepancy remains an important one to note.

Given the commissioning of SSSs was not the sole responsibility for the vast majority of respondents; commissioners may lack sufficient time within their role to provide large amounts of detail on how services should be managed. The mean length of time spent commissioning health services was also greater than that spent commissioning stop smoking services, meaning that many of these commissioners may have had smoking cessation added to a broader portfolio of services to commission, and may not have sufficiently high levels of expertise to make specific recommendations.

Despite these issues, the majority of managers indicated that the treatments and services they offered were in line with evidence based guidelines. The most commonly offered treatment model was one-to-one, which is in line with SSS statistics showing that most smokers set a quit date using this model in 2009/2010 [9]. Although systematic review evidence indicates that one-to-one support for smoking cessation is of proven efficacy when compared with self-help interventions, group interventions have shown a bigger effect [13-15] and their superior efficacy is reported in current SSS guidelines [6]. Although only 2% of smokers set a quit date using a closed group programme in 2009/2010 [9], over two thirds of managers indicated that they offered this treatment model. Previous research [8] has suggested that the shift towards one-to-one services was largely due to client preference for one-to-one services, and this may well account for some of the discrepancy between the availability and uptake of other, more effective treatment models such as closed groups. Further examination of why so few smokers take up this treatment option despite its availability may be worthy of further attention.

The majority of SSS managers also indicated that all NICE-approved medications (NRT, bupropion & varenicline) were available as first-line treatments. Improved treatment outcomes could still be achieved, however, were evidence-based guidelines adhered to more stringently. Based on managers’ reports of the availability of the most effective treatments and current rates of uptake by clients and effectiveness at NHS SSSs [9], had 100% of managers made closed groups available to SSS attendees in 2009/2010 it could have led to 4,610 extra quitters that year. Similarly, had 100% of SSS managers made varenicline available it could have resulted in 17,249 additional quitters.

### Future research

The current study suggests several avenues for further research. Of primary importance is to explore managers’ and commissioners’ perceptions of whose responsibility it is to ensure that evidence-based guidelines are applied in practice. Future research questions could also explore the gaps between managers’ reported availability of the most effective treatments, i.e. closed groups, and the observed uptake in SSS statistics [9]. As well as encouraging SSS managers to increase availability of these interventions it may also be important to further examine why so few smokers take up this treatment option despite its availability and to intervene if necessary. For commissioners, future research could include analysis of SSS specifications, including their content and the level of detail included, investigation of commissioners’ perspectives on their roles and responsibilities, and commissioner confidence in whether SSS are compliant with service specifications and whether this confidence is reflected in the level of active involvement they have with service delivery. It would also be of great interest to investigate whether these aspects of commissioners’ and managers’ practices are associated with abstinence rates.

### Strengths & limitations

The response rate for both surveys was 35%, which although lower than previous research of SSS managers [22,23] is comparable with the average response rate of 40% reported by Cook and colleagues [24] in a meta-analysis of 68 studies based on online surveys. The PCTs from which commissioners and managers responded did not, however, appear to differ significantly from those PCTs from which no one responded both in terms of local deprivation, or SSS outcome or throughput. Another limitation is the amount of missing data in the survey, which was as much as 28% for some items. Mandatory fields could be used to minimise this in future surveys. However, conversely, one strength of the survey was the inclusion of multiple free-text response options, meaning that respondents were not constrained by the options pre-determined by the researchers using fixed, tick-box questions, allowing them more freedom of expression.

## Conclusions

Whilst the majority of managers indicated that the treatments and services they offered were in line with evidence based guidelines, treatment outcomes for smokers could be improved were guidelines adhered to more stringently. A substantial part of commissioning of Stop Smoking Services in England appears to take place without adequate consultation of evidence-based guidelines or specification of the service to be provided. Although commissioners may allow SSS managers the autonomy to configure their own services in the expectation that guidelines are adhered to, the current surveys indicate that they may need to clearly and explicitly refer to the evidence base in service specifications to ensure that smokers have access to the most effective interventions. The current failure to achieve this level of specification may account for at least some of the variation in success rates. Future research should attempt to ascertain whose responsibility commissioners and managers perceive it is to ensure that guidelines for best practice are adhered to. Work could then be conducted to establish a clear procedure for the translation of the evidence base into practice.

## Misc

Heather Thomson, Robert West, Jennifer AM Kenyon and Andy McEwen contributed equally to this work

## Competing interests

MSMcD is employed by the NHS Centre for Smoking Cessation and Training (NCSCT). HT has received hospitality from Pfizer, who manufacture Champix. She is employed by NHS Leeds who have been recipients of research funds from the National Institute of Health Research. RW is a director of the NCSCT. He undertakes research and consultancy for companies that develop and manufacture smoking cessation medications (Pfizer, J&J, McNeil, GSK, Nabi, Novartis and Sanofi-Aventis). He also has a share of a patent for a novel nicotine delivery device and is a trustee of QUIT, a charity that provides stop smoking support. JK has received hospitality from Pfizer, who manufacture Champix. AMcE is also a director of the NCSCT. He has received travel funding, honoraria and consultancy payments from manufacturers of smoking cessation products (Pfizer, J&J, McNeil, GSK, Nabi, Novartis and Sanofi-Aventis). He also receives payment for providing training to smoking cessation specialists, receives royalties from books on smoking cessation and has a share in a patent of a nicotine delivery device.

## Authors’ contributions

MSMcD collected, cleaned and analysed the data, drafted and revised the paper. HT contributed to the data analysis, drafted and revised the paper. RW & JK revised the draft paper. AMcE secured funding for this study and revised the draft paper. All authors had full access to all of the data (including statistical reports and tables) in the study and can take responsibility for the integrity of the data and the accuracy of the data analysis. All authors approved the final version of the article.

## Pre-publication history

The pre-publication history for this paper can be accessed here:

http://www.biomedcentral.com/1472-6963/12/121/prepub

## Supplementary Material

Additional file 1 Study Questionnaires (Appendix A & Appendix B) (Additional material Study questionnaires.doc). Click here for file
