# Supplementary material for: Translating evidence-based guidelines into practice: a survey of practices of commissioners and managers of the English stop smoking services
Source: BMC Health Serv Res. 2012 May 23;12:121. doi: 10.1186/1472-6963-12-121 (PMC3444944; doi:10.1186/1472-6963-12-121)
Supplement: Additional file 1 — Study Questionnaires (Appendix A & Appendix B) (Additional material Study questionnaires.doc). [file 1472-6963-12-121-S1.docx]

# Additional material: Study questionnaires

# Appendix A: Commissioners’ survey

# NCSCT Annual Survey of Commissioners of Stop Smoking Services: 2010

| The NHS Centre for Smoking Cessation and Training (NCSCT) has been commissioned by the Department of Health to provide training and resources for those that deliver, manage and commission Stop Smoking Services. This is the second annual survey of smoking cessation commissioners conducted by the NCSCT, which gives you the chance to give us an up to date view of the issues, barriers and systems that are important in your area and will help us to plan the delivery of NCSCT services over the coming years for maximum benefit.  The survey should take no more than 15 minutes to complete. Questionnaire responses and any other information given during the course of the research will be anonymous. All information will be used for research purposes only. Please note that confidentiality will be maintained and it will not be possible to identify you from any report about this study.  For more information on the work of the NCSCT see: www.ncsct.co.uk |
| --- |

**Contact details:**

Note: This information will not be passed onto anyone but will ensure that we have your correct details and will allow us to contact you in the future about resources and training that may be relevant to your needs.

| Name: |  |
| --- | --- |
| Job title: |  |
| PCTs you are responsible for (please list all): |  |
| Email address: |  |
| Telephone number: |  |
| Postal address: |  |

**Some questions about the commissioning of stop smoking services:**

| Are Stop Smoking Services commissioned solely by the Primary Care Trust? | Yes | No |
| --- | --- | --- |
| If not, please state how the services are commissioned? (please write in box) |  | |
| Is commissioning of the Stop Smoking Service based on a local needs assessment? | Yes | No |
| If so, what information does this needs assessment contain? (please write in box) |  | |
| Beyond the local needs assessment what evidence has informed local commissioning? (please write in box) |  | |
| Is smoking cessation (4 week quit rates)/ reduction in smoking prevalence included in the PCT’s priorities? | Yes | No |
| Are stop smoking services commissioned from a single provider organisation? | Yes | No |
| Are stop smoking services commissioned from an NHS provider organisation? | Yes | No |
| Has a commissioning framework been developed that takes various elements of stop smoking support (e.g. very brief advice through to intensive support) and different settings for provision into account? | Yes | No |

| Do you include, within your service specification, targets for? (please tick all that apply): | | |
| --- | --- | --- |
| Achieving 4 week quit rates | |  |
| Achieving carbon monoxide (CO) validation rates (minimum of 85%) | |  |
| Throughput of smokers from Routine and Manual groups (minimum of 50%) | |  |
| Reducing prevalence of smoking during pregnancy | |  |
| Reducing local smoking prevalence | |  |
| Reducing or contributing towards reducing in health inequalities | |  |
| Working with locally identified target groups | |  |
| Other (please write in box) | |  |
| Do you specify the types of stop smoking services (e.g. 1:1, groups, home visits) to be delivered by the providers? | Yes | No |
| Do service specifications for smoking state the level of training that advisors are expected to achieve? | Yes | No |
| Do service specifications for smoking state requirements for the continuing professional development of staff? | Yes | No |

**Some questions about prescribing at your stop smoking service:**

| What medications are available through your service as a 1^st^ line of treatment: |  | |
| --- | --- | --- |
| NRT | Yes | No |
| Bupropion (Zyban) | Yes | No |
| Varenicline (Champix) | Yes | No |

**Some questions about you:**

| Are you: | | | Male | Female | | No answer |
| --- | --- | --- | --- | --- | --- | --- |
| How old are you? (please write age in box) | | | | | ……Years | |
| Which Directorate or Department currently employs you? (please write in box) | | | | |  | |
| How long have you been commissioning **health services**? (please write in box) | | | | | .…..Years.…..Months | |
| How long have you been commissioning **stop smoking services**? (please write in box) | | | | | .…..Years.…..Months | |
| Can you estimate what percentage of your current role involves the commissioning of **stop smoking services**? (please write in box) | | | | | % | |
| What **other services**, if any, do you also have responsibility to commission? (please write in box and estimate proportion of time spent on this) |  | | | | % | |
|  |  | | | | % | |
|  |  | | | | % | |
|  |  | | | | % | |
|  |  | | | | % | |
|  |  | | | | % | |
| How many staff do you have working for you? (please write in box) | | .…..Full time | | | .…..Part time | |

# Appendix B: Managers’ survey

# NCSCT Annual Survey of Managers of Stop Smoking Services: 2010

| The NHS Centre for Smoking Cessation and Training (NCSCT) has been commissioned by the Department of Health to provide training and resources for those that deliver, manage and commission Stop Smoking Services. This is the second annual survey of smoking cessation managers conducted by the NCSCT, which gives you the chance to give us an up to date view of the issues, barriers and systems that are important in your area and will help us to plan the delivery of NCSCT services over the coming years for maximum benefit.  The survey should take no more than 15 minutes to complete. Questionnaire responses and any other information given during the course of the research will be anonymous. All information will be used for research purposes only. Please note that confidentiality will be maintained and it will not be possible to identify you from any report about this study.  For more information on the work of the NCSCT see: www.ncsct.co.uk |
| --- |

**Contact details:**

Note: This information will not be passed onto anyone but will ensure that we have your correct details and will allow us to contact you in the future about resources and training that may be relevant to your needs.

| Name: |  |
| --- | --- |
| Job title: |  |
| Name of Stop Smoking Service: |  |
| Employing organisation: |  |
| Does your service provide stop smoking services to any other PCTs? (please list) |  |
| Email address: |  |
| Telephone number: |  |
| Postal address: |  |

**Some questions about you:**

| Are you: | | Male | Female | | No answer |
| --- | --- | --- | --- | --- | --- |
| How old are you? (please write age in box) | | | | ……Years | |
| How long have you been managing stop smoking services? (please write in box) | | | | .…..Years  .…..Months | |
| Can you estimate what percentage of your current role involves the managing of your stop smoking service? (please write in box) | | | | % | |
| What **other services**, if any, do you also have responsibility to manage? (please write in box and estimate the proportion of your time spent on this) |  | | | % | |
|  |  | | | % | |
|  |  | | | % | |
|  |  | | | % | |
|  |  | | | % | |
|  |  | | | % | |

**Some questions about your service:**

| Please indicate whether your service offers the following treatment models: | | | |
| --- | --- | --- | --- |
| One-to-one appointments | Yes | | No |
| One-to-one drop-in sessions | Yes | | No |
| Closed group programmes | Yes | | No |
| Rolling group programmes | Yes | | No |
| Telephone advice/counselling | Yes | | No |
| Home visits | Yes | | No |
| Self-help materials | Yes | | No |
| Peer led sessions | Yes | | No |
| Other: | Yes | | No |
| Other: (please write in box) |  | | |
| Does the commissioner of the stop smoking service specify the type of treatment models to be offered? | Yes | No | |
| Please indicate in which settings your services run: | | | |
| Central base exclusive to SSS | Yes | No | |
| Primary care settings | Yes | No | |
| Secondary health care settings (e.g. hospitals) | Yes | No | |
| Commercially rented venues | Yes | No | |
| Voluntary sector/Local Authority premises | Yes | No | |
| Pharmacies | Yes | No | |
| Work places | Yes | No | |
| Other: | Yes | No | |
| Other: (please write in box) |  | | |
| Does the commissioner of the stop smoking service specify the settings in which the service should run? | Yes | No | |

| How many Whole Time Equivalent (WTE) stop smoking practitioners do you currently employ where providing stop smoking services is the main part of their job? (please write in box) |  | |
| --- | --- | --- |
| Is the stop smoking service you manage responsible for the support/ performance management of other practitioners who incorporate smoking cessation as part of their wider role (e.g. health visitors, practice nurses…)? | Yes | No |
| How many of these other practitioners who incorporate smoking cessation as part of their wider role do you support/ performance manage? (please write in box) | |  |
| What proportion of other practitioners who incorporate smoking cessation as part of their wider role do you estimate are ‘active’ ( ie have seen over 5 clients in the last 6 months and returned monitoring forms) (please write in box) | | % |

| **Do you have a dedicated member of staff who leads on providing a service to:** | Yes | No |
| --- | --- | --- |
| Pregnant smokers |  |  |
| Young smokers |  |  |
| Smokers with mental health problems |  |  |
| Prisoners |  |  |
| Smokers from routine and manual groups |  |  |
| Smokers from black and minority ethnic groups |  |  |
| Smokers in the workplace |  |  |
| Smokers in hospital |  |  |
| Other (please list): |  |  |

| Does your service have specific targets within the service specification around (please select all that apply): | | |
| --- | --- | --- |
| Achieving 4 week quit rates targets | |  |
| Carbon monoxide (CO) validation rates (minimum of 85%) | |  |
| Throughput of smokers from Routine and Manual groups (minimum of 50%) | |  |
| Reducing local smoking prevalence | |  |
| Reducing or contributing to reducing health inequalities | |  |
| Reducing prevalence of smoking during pregnancy | |  |
| Working with locally identified target groups, e.g. pregnancy, BME, R&M, etc | |  |
| Other (please write in box) | |  |
| Do you have regular arranged meetings with your commissioner? | Yes | No |
| Do you think that you have a good relationship with your commissioner? | Yes | No |

**Some questions about the training your staff receives?**

| How many days theoretical training do your stop smoking practitioners receive when they join your team? (Please write number in box) | ………….. days | |
| --- | --- | --- |
| Do your stop smoking practitioners observe an experienced practitioner before seeing clients on their own? (Please indicate the number of days in box) | ………….. days | |
| Is the stop smoking service responsible for providing training to other practitioners who incorporate smoking cessation as part of their wider role? | Yes | No |

**Some questions about prescribing at your stop smoking service:**

| What medications are available through your service as a 1st line of treatment: |  | |
| --- | --- | --- |
| NRT | Yes | No |
| Bupropion (Zyban) | Yes | No |
| Varenicline (Champix) | Yes | No |
